# Unique genetic responses revealed in RNA-seq of the spleen of chickens stimulated with lipopolysaccharide and short-term heat

**DOI:** 10.1371/journal.pone.0171414

**Published:** 2017-02-06

**Authors:** Angelica Van Goor, Chris M. Ashwell, Michael E. Persia, Max F. Rothschild, Carl J. Schmidt, Susan J. Lamont

**Affiliations:** 1 Department of Animal Science, Iowa State University, Ames, IA, United States of America; 2 Department of Poultry Science, North Carolina State University, Raleigh, NC, United States of America; 3 Department of Animal and Poultry Sciences, Virginia Polytechnic Institute and State University, Blacksburg, VA, United States of America; 4 Department of Animal and Food Sciences, University of Delaware, Newark, DE, United States of America; University of Florida, UNITED STATES

## Abstract

Climate change and disease have large negative impacts on poultry production, but little is known about the interactions of responses to these stressors in chickens. Fayoumi (heat and disease resistant) and broiler (heat and disease susceptible) chicken lines were stimulated at 22 days of age, using a 2x2x2 factorial design including: breed (Fayoumi or broiler), inflammatory stimulus (lipopolysaccharide (LPS) or saline), and temperature (35°C or 25°C). Transcriptional changes in spleens were analyzed using RNA-sequencing on the Illumina HiSeq 2500. Thirty-two individual cDNA libraries were sequenced (four per treatment) and an average of 22 million reads were generated per library. Stimulation with LPS induced more differentially expressed genes (DEG, log_2_ fold change ≥ 2 and FDR ≤ 0.05) in the broiler (N = 283) than the Fayoumi (N = 85), whereas heat treatment resulted in fewer DEG in broiler (N = 22) compared to Fayoumi (N = 107). The double stimulus of LPS+heat induced the largest numbers of changes in gene expression, for which broiler had 567 DEG and Fayoumi had 1471 DEG of which 399 were shared between breeds. Further analysis of DEG revealed pathways impacted by these stressors such as Remodelling of Epithelial Adherens Junctions due to heat stress, Granulocyte Adhesion and Diapedesis due to LPS, and Hepatic Fibrosis/Hepatic Stellate Cell Activation due to LPS+heat. The genes and pathways identified provide deeper understanding of the response to the applied stressors and may serve as biomarkers for genetic selection for heat and disease tolerant chickens.

## Introduction

Climate change will increase the degree and frequency of severe weather patterns, and the global average temperature is expected to become increasingly warmer [1], which will have large negative impacts on poultry production [2]. Heat stress generally decreases immunocompetence in chickens, characterized by decreased relative weights of immune tissues [3, 4, 5], decreased antibody production [6, 7], increased incidence of bacterial colonization of the spleen [8], higher susceptibility to infections [9], and decreased macrophage activity [3]. St-Pierre estimated $58 million dollars in production losses annually in poultry due to heat stress in the U.S. alone [2], and disease is estimated to cause 20% of production losses in the poultry industry [10], and is a concern for animal welfare and human health.

Several studies have identified decreases in adaptive immunity in chickens during heat stress conditions. A 5-week period of heat stress in layers decreased total white blood cell count, antibody production, and lymphocytes activity [6]. Examination of the intestinal histology in laying hens during heat stress identified an increase in the number of intraepithelial lymphocytes [11]. Broilers that are heat stressed have lower weights of immune organs including the bursa of Fabricius, thymus, and spleen [3, 4, 5]. Lower relative weight in lymphoid organs may indicate immunosuppression [12], and has been associated with decreased immune response to Newcastle Disease in chickens [13]. Total circulating antibody is decreased in broilers under heat stress [5]. When broiler chicken lines selected for high and low antibody titre to SRBC were subjected to heat stress, the highly responsive line had decreased antibody production to SRBC under heat stress conditions compared to thermoneutral conditions [7]. In addition to adaptive immune cell reaction to heat stress, broilers have increased susceptibility to mild enteritis characterized by increased concentrations of white blood cells in lamina propria of the jejunum [8]. On the contrary to immunosuppressive effect, short bouts (1–2 hours) of heat stress have been shown to increase antibody production to SRBC in broilers [14]. Macrophages have lower basal and bacterial induced oxidative burst activity during heat stress [3]. Broilers heat stressed and challenged with *Salmonella enterica* serovar Enteritidis have increased bacterial invasion of the spleen, and the authors speculate this may be due to gut barrier dysfunction during heat stress (8). Broilers under heat stress conditions increase intestinal permeability [8, 3, 15], and layers have altered gut morphology of microvilli [11]. In humans, increase intestinal permeability causes a rapid increase in bacteria within the blood, which can lead to endotoxic shock, sepsis, and death largely due to a pro-inflammatory cytokine storm [16]. The major contributing factor to the cytokine storm caused by disruption of the gut barrier is thought to be lipopolysaccharide (LPS) [17]. LPS is an essential component of gram negative bacteria and a major contributor to the fatality of heat stroke in humans [18]. The double stimulation of LPS and heat stress could increase body temperatures beyond the thermal comfort zone, resulting in increased mortality. However, the type of stressor and the time of exposure determines the immune response [19].

Chicken breeds with distinct genetic background, such as Fayoumi and broiler, may represent different levels of adaptation to pathogenic and environmental stressors, and serve as an excellent discovery platform to investigate genetic differences in the stress response. The broiler breed was commercially selected for muscle mass accretion, whereas Fayoumi represents a wild-type strain of chicken, originated in Egypt. Fayoumi is a hardier genetic line than broiler with a higher level of heterophil response to *Salmonella Enteritis* [20] compared to broilers. The spleen was chosen for study to determine the effect of the double stimulus (heat and LPS) on the immune system. The spleen functions as a major site of response to infection by harboring immune cells such as macrophages, B cells and T cells in close proximity, which encounter pathogens circulating in the blood, which in turn initiate an immune response. We hypothesize that heat stress would result in a suppressed immune response to LPS and that the spleen, as a major immune effector organ, is an important site for modulating that response. To our knowledge, the spleen in chicken has never been tested for response to heat stress and immune stimulation in vivo. In the current study, the spleen transcriptome was sequenced to investigate the effect of LPS, heat stress, and LPS+heat.

## Materials and methods

### Ethics statement

All animal experiments were approved by the Institutional Animal Care and Use Committee at Iowa State University: Log #4-11-7128-G.

### Experimental design and tissue sampling

Chicks were produced from the same breeders in two hatches (replicates). The Fayoumi (heat and disease resistant) and the broiler (heat and disease susceptible) breeds were assessed for response to heat stress and LPS stimulation using a full factorial design including the factors; breed, thermal treatment, and inflammatory stimulus. A total of 32 chickens (4 chicken spleen per treatment) were used for RNAseq. At 17 days of age, birds were transferred to environmentally-controlled chambers and acclimated for five days. The multiple environmentally-controlled chambers each contained 6 pens of one-meter square in which the birds were housed. The two breeds were in separate pens, but each chamber contained both breeds. The temperature was 25°C, except during the heat stress period (raised to 35–37°C). Humidity was monitored, but not controlled during the experiment. Floor pens had wood shavings for bedding and birds had *ad libitum* access to water and corn-soy feed that met all NRC requirements for the duration of the study [21]. There were four environmental chambers, each containing four pens, per replicate. One to two chickens were sampled per pen per replicate.

At 22 days of age, two of the chambers heated to 35°C (heat stress, HS), while the other two remained at 25°C (thermoneutral, TN). After 3.5 hours of thermal treatment, half of the birds were subcutaneously injected with LPS (LPS) (Sigma-Aldrich L7261, *Salmonella enterica* serotype typhimurium) in the amount 100 μg/kg of body weight, or phosphate buffered saline (PBS). This design resulted in 8 treatment groups: 1. Fayoumi, TN, PBS (F_TN_PBS); 2. Fayoumi, HS, PBS (F_HS_PBS); 3. Fayoumi, TN, LPS (F_TN_LPS); 4. Fayoumi, HS, LPS (F_HS_LPS); 5. Broiler, TN, PBS (B_TN_PBS); 6. B, HS, PBS (B_HS_PBS); 7. Broiler, TN, LPS (B_HS_LPS) 8. Broiler, HS, LPS (B_HS_LPS). After a total of 7 hours of heat stress (3.5 hours post LPS stimulation) chickens were humanely euthanized with intravenous injection of a lethal dose of Sodium Pentobarbital (Sleepaway, Fort Dodge Animal Health). The spleens were immediately harvested, placed in RNAlater, and stored at -80°C until further use.

The sex of the animals was determined post-euthanasia and determined 16 males, 14 females, and 2 chickens of undetermined sex (data not recorded) were used for RNA-sequencing. Within each treatment at least 1 male was used.

### Body temperature and blood chemistry component measurements

Cloacal body temperature was measured by inserting a digital thermometer approximately 2.5 cm into the cloaca on days 20 (pre-treatment) and 22 of age (at the end of treatment). The precision of the digital thermometer was 0.1°C. Blood was collected from the wing vein on day 20 (pre-heat) and day 22 (at the end of treatment after body temperature was measured) using a heparinized syringe and needle, and analysed immediately using an iSTAT Portable Clinical Analyser [22]. The iSTAT CG8+ cartridge was utilized to measure thirteen blood variables including; pH, pCO_2_, pO_2_, base excess, HCO_3_, TCO_2_, K, Na, ionized Ca, hematocrit, hemoglobin, sO_2_, and glucose.

### Library generation and sequencing

The total RNA was isolated from 4 chicken spleens per treatment group (32 birds total) with the RNAqueous kit (Ambion) using all manufacturers recommendations as previously described [23], and treated with DNA-free kit (Ambion). The RNA quality was quantified using the Agilent 2000 Bioanalyzer. Only samples with an RNA Integrity Number (RIN) greater than 9 were used for cDNA library construction using the TruSeq RNA Library preparation kit v2 (Illumina) that preferentially amplifies PolyA mRNA. The cDNA libraries created from the spleen of 32 chickens were run on the HiSeq 2500 machine (Illumina) with the option of 100 bp single end reads at the Iowa State University DNA Facility. Four lanes were used for RNA-sequencing with 8 multiplexed samples per lane (one sample per each treatment in each lane).

### Detection of differential gene expression and average sequencing depth analysis

Quality control of RNA-seq reads was conducted using FASTQC and FASTX Clipper (version 0.0.13; http://hannonlab.cshl.edu/fastx_toolkit/) with the following options; phredd score of 30, minimum base pair length of 30, and adapter sequences were removed.

Reads were mapped to the *Gallus gallus* genome version 4.0 (4.78 GTF Ensembl) using Tophat (version 2.0.9) [24] using default parameters. Counting of mapped reads to a gene was done using HTSeq (version 3.0). Table 1 includes the average (N = 4/treatment group) number of generated reads before and after quality filtering, number of mapped and the percentage of transcriptome coverage. There are 15,508 annotated coding genes in *Gallus gallus* 4.0 genome. To calculate transcriptome coverage, we used the average number of annotated genes that were expressed in our dataset and divided by the number of annotated coding genes. The resulting number is within range of predicted coverage based on sequencing depth [25]. Differential gene expression was detected using EdgeR (version 1.00), with Benjamini Hochberg method used for multiple testing correction, and maximum FDR ≤ 0.05. A pairwise comparison was used to detect DEG within breed by contrasting each treatment with the most naive group, i.e. TN_PBS. The contrasts contained 4 individuals per treatment group and were as follows; F_TN_PBS vs F_HS_PBS, F_TN_PBS vs F_TN_LPS, F_TN_PBS vs F_HS_LPS, B_TN_PBS vs B_HS_PBS, B_TN_PBS vs B_TN_LPS, and B_TN_PBS vs B_HS_LPS.

**Table 1:** Average number of RNA-sequencing reads generated for each chicken spleen within each treatment group (N = 4) before and after FASTQC filtering, and number of mapped reads, and percentage of sequencing depth analysis. Intragenic regions are considered reads that mapped to all or part of an exon and therefore included as feature counts in HTSeq, whereas intergenic regions are reads that mapped outside of regions annotated as exons.

Treatment group	Reads generated	Reads post-filtering	Mapped reads (%)	Transcriptome coverage (%)	Intragenic mapping (%)	Intergenic mapping (%)
F_TN_PBS	24,200,942	19,841,278	17,480,166 (88.1%)	12,987 (83.7%)	89.3	10.7
F_HS_PBS	20,203,988	16,721,600	14,915,667 (89.2%)	12,885 (83.1%)	88.7	11.3
F_TN_LPS	28,607,883	23,464,251	21,023,969 (89.6%)	12,534 (80.8%)	89.2	10.8
F_HS_LPS	22,828,621	18,843,821	20,568,587 (90.1%)	12,717 (82.0%)	88.1	11.9
B_TN_PBS	17,551,738	14,404,416	12,863,143 (89.3%)	12,692 (81.8%)	89.6	10.4
B_HS_PBS	18,184,950	14,859,218	13,239,563 (89.1%)	12,568 (81.0%)	89.5	10.5
B_TN_LPS	19,724,297	16,050,493	14,236,787 (88.7%)	12,675 (81.7%)	88.3	11.7
B_HS_LPS	20,720,682	16,978,131	15,059,602 (88.7%)	12,632 (81.5%)	88.5	11.5
Average	21,502,888	17,645,401	16,173,436 (89.1%)	12,711 (82.0%)	88.9	11.1

### Fluidigm expression verification of RNA-seq data

We used Fluidigm gene expression technology to confirm the mRNA expression detected by RNA-seq in the current study. As described in the Library Generation and Sequencing section, total RNA was isolated and treated with a DNA-free kit. Gene expression analysis was performed using a microfluidic Reverse Transcription quantitative PCR (RT-qPCR) (Fluidigm Corporation, San Francisco, CA, USA). All procedures were conducted according to manufacturer’s recommendations, unless otherwise noted. Briefly, 50 ng of the total RNA was reverse transcribed using the Fluidigm Reverse Transcription Master Mix (Fluidigm Corporation, San Francisco, CA, USA). cDNA was pre-amplified with PreAmp Master Mix (Fluidigm Corporation, San Francisco, CA, USA), using 12 cycles of pre-amplification. Exonuclease I (New England Biolabs, UK) treatment was applied to remove unincorporated primers. Pre-amplified and purified cDNA samples were diluted 10x in TE buffer and stored at -20°C until further analyses. RT-qPCR analysis was completed for 22 target genes and 2 reference genes, listed in S1 Table. These genes were selected to assay because they represented a range of fold change expression values from extremely changed (LFC ≥ ±30) to not changed due to treatment (LFC = 0). The FlexSix Integrated Fluid Circuits (IFCs) (Fluidigm Corporation, San Francisco, CA, USA) was used to assay mRNA expression. Sample assay included 1.35 μl of pre-amplified and Exo I treated cDNA, 1.5 μl of the SsoFast™ EvaGreen^®^Supermix with Low ROX™ (2x) (Bio-Rad) and 0.15 μl of the FlexSix Delta Gene Sample Reagent (Fluidigm Corporation, San Francisco, CA, USA). Primer assays were prepared as 20 μl stock by mixing 1μl of each primer (100 μM) with 10 μl of the 2x Assay Loading Reagent and adjusted to 20 μl with DNA suspension buffer (low EDTA TE buffer). The samples, assays and the loading reagents were then loaded onto IFCs microfluidic channels using the RX loading station (Fluidigm Corporation, San Francisco, CA, USA). RT-qPCR was performed on the Biomark™ HD (Fluidigm Corporation, San Francisco, CA, USA) using the fast program that consisted of an incubation step at 95°C for 60 s followed by 30 cycles: 96°C for 5s and 60°C for 20s. Fluorescence emission was recorded after each cycling step. Upon RT-qPCR completion, melting curves were generated by increasing temperature from 60 to 95°C, with continuous fluorescence acquisition.

RT-qPCR data were analyzed as follows: raw qPCR data were analyzed and checked for quality using Real-Time PCR Analysis Software (Fluidigm Corporation, San Francisco, CA, USA). Main effects of the stimulation of the spleen were estimated using least square means method implemented in JMP Pro 10.0.2 software (SAS Institute, Cary, NC, USA). Chicken line (Fayoumi or broiler), thermal treatment (TN or HS), and immune stimulus (PBS and LPS) as well as the interaction between line and treatments were fitted in the model. Analyses were performed separately for each line, gene, and treatment using dCt values (Ct target–Ct reference). To determine the relative gene expression, ddCt method was used [26]. Delta Ct values were obtained by normalizing the Ct values of the target genes with the geometrical mean of the two reference genes (*H6PD* and *RPL4*). Fold induction of the gene expression was estimated as 2^-ddCt^. Untreated (control) samples were used as calibrators.

## Data deposition

The data discussed in this publication is deposited in the Gene Expression Omnibus at NCBI [27] with the GEO accession number GSE85434.

## Results

### Body temperature and blood chemistry component response

**Fig 1:**
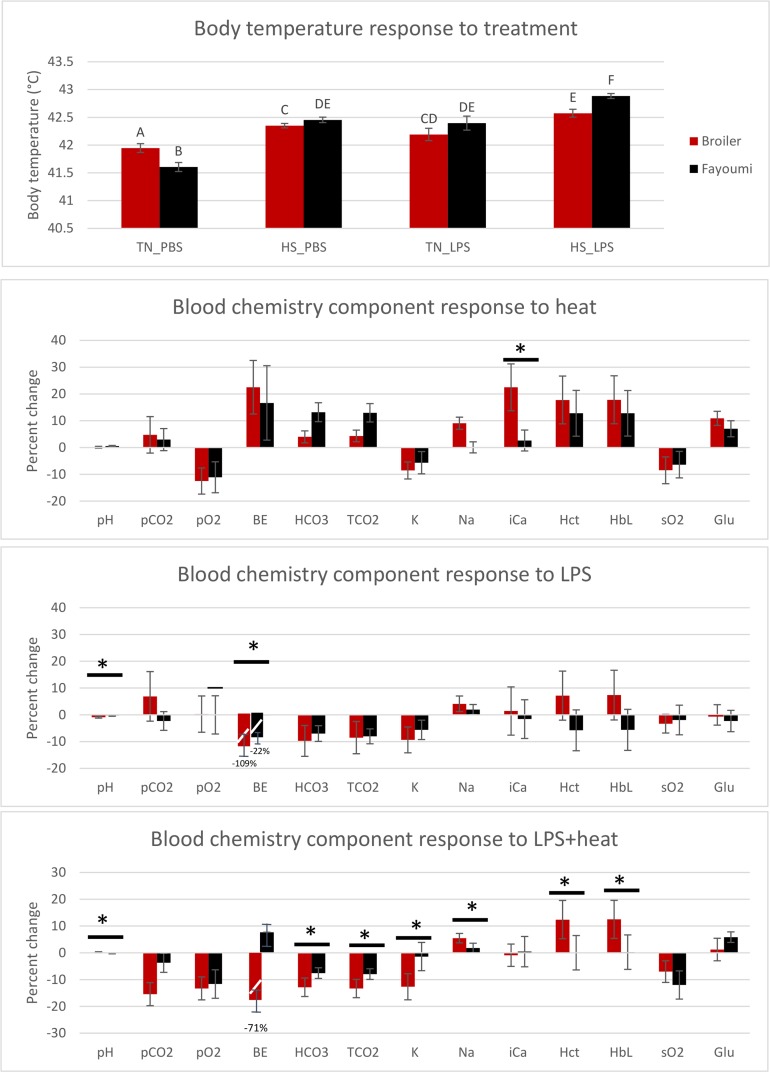
Phenotypic responses to treatment in the Fayoumi and broiler chicken breeds at 22 days of age after 7 hours of heat treatment at 35°C, and/or 3.5 hours post lipopolysaccharide (LPS) treatment. Cloacal body temperature and blood chemistry components measured using an iSTAT machine. Chicken breeds are depicted by different colors with broiler in red and Fayoumi in black. A. Body temperature response to treatments. B. Blood chemistry component changes in response to heat, C. LPS, and D. LPS+heat. * indicates significant pairwise difference between breeds (P ≤ 0.05).

The body temperature and blood chemistry results are presented in Fig 1. Body temperature significantly (P ≤ 0.05) increased in both breeds in response to the single treatments of heat or LPS, and the increases were of the same magnitude for each treatment. The double stimulus of LPS+heat resulted in a synergistic increase; i.e., it was statistically (P ≤ 0.05) higher than the single treatments. Blood chemistry components are presented as percent change from pre-heat values. Between-breed comparisons reveal relatively few (10 with P ≤ 0.05) differences, possibly because of the large variability. The double stimulus of LPS+heat resulted in the most differences between breeds.

### Alignment and mapping to the chicken genome

The RNA-seq generated from 32 individual cDNA libraries (4 per treatment group) as reported in Table 1 were mapped to *Gallus gallus* 4.0. Across all treatment groups, the average number of reads generated per cDNA library was 21,502,88; post filtering resulted in 17,645,401; the percentage of reads that mapped to the chicken genome was 89%; and the percentage of the spleen transcriptome that was expressed was 82%. The cutoff value for expressed genes was 1 FPKM.

### Differentially expressed genes

**Fig 2:**
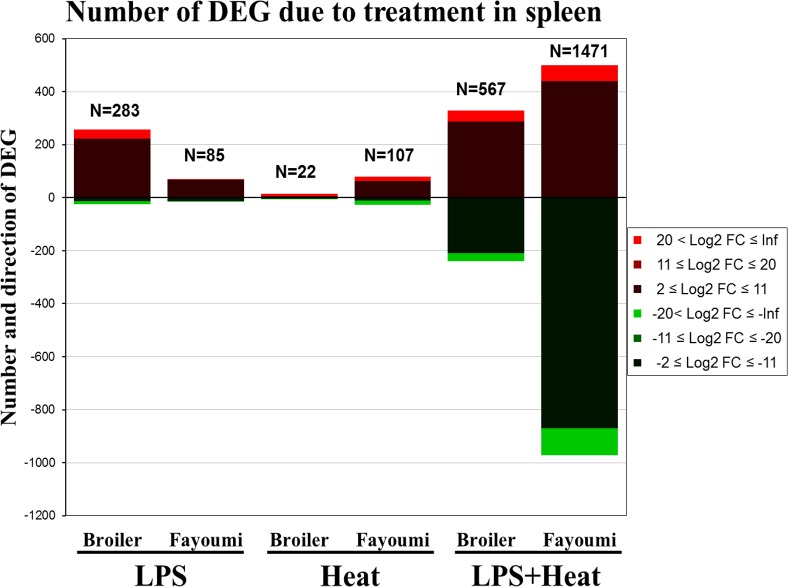
A heat map showing the number of differentially expressed genes (DEG) and the direction of log fold change (LFC) of expression values in the spleen of the Fayoumi and broiler chicken breeds at 22 days of age after 7 hours of heat treatment at 35°C, and/or 3.5 hours post lipopolysaccharide (LPS) treatment. DEG have an FDR ≤ 0.05 and LFC ≥ ± 2.

**Fig 3:**
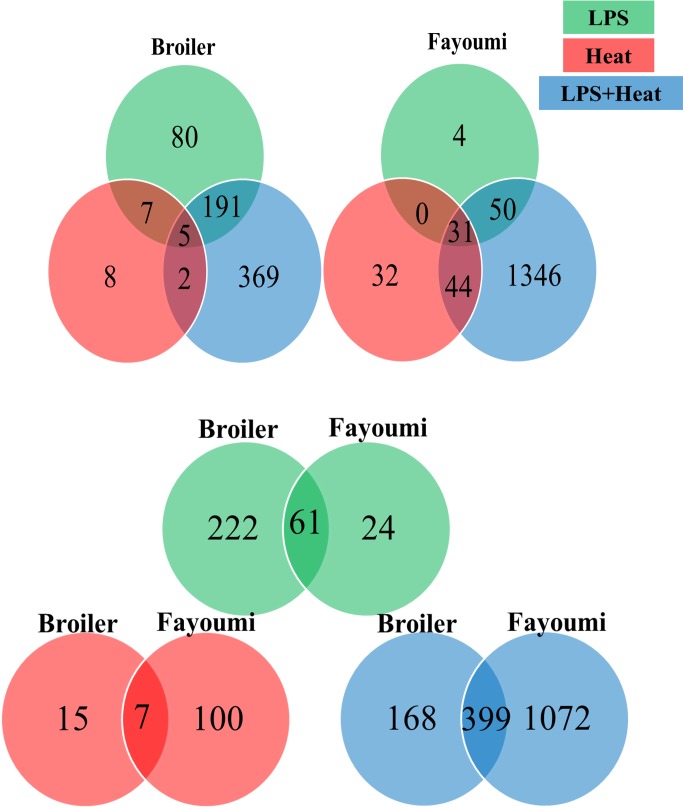
Venn diagrams depicting shared differentially expressed genes between treatments and chicken breeds. Fayoumi and broiler chicken breeds at 22 days of age after 7 hours of heat treatment at 35°C, and/or 3.5 hours post lipopolysaccharide (LPS) treatment. Green is treatment with LPS, red is heat, and blue is LPS+heat. Genes were considered differentially expressed with LFC ≥ ± 2 and FDR ≤ 0.05.

Differentially expressed genes (DEG) within the spleen were detected using EdgeR software with a LFC ≥ ± 2 and FDR ≤ 0.05 and are depicted in Fig 2 as a heat map that indicates total number of DEG as well as the direction of fold change. Broilers responded to LPS (B_TN_PBS vs B_TN_LPS) by 283 DEG, with only 25 (less than 10%) downregulated. Fayoumis responded to LPS (F_TN_PBS vs F_TN_LPS) by 85 DEGs, with more than 80% upregulated. Broilers responded to thermal treatment (B_TN_PBS vs B_HS_PBS) by only 22 DEG, with 15 upregulated and 7 downregulated. Fayoumis responded to thermal treatment (F_TN_PBS vs F_HS_PBS) with almost 5 times more (107) DEG compared to broilers, with nearly two-thirds (79) upregulated and one-third (28) downregulated. Broilers responded strongest to LPS+heat (B_TN_PBS vs B_HS_LPS) treatment by 567 DEG, with the majority (328) upregulated and 239 downregulated. The contrast which resulted in the most DEG was in Fayoumis that responded to treatment with LPS+heat (F_TN_PBS vs F_HS_LPS) by 1471 DEGs, and nearly twice as many were downregulated (972) than upregulated (499). The shared DEGs within breed between treatment groups, and the shared DEGs between breeds within treatment groups are depicted as Venn Diagrams in Fig 3. Within-breed comparison for DEG due to treatment reveal that there are few genes in common between LPS and heat (Fayoumi 0 and broiler 7). Also, the Fayoumi has 6 times (31 DEG) more genes that are shared between all three treatments than the broiler (5 DEG). Comparing between lines within treatments, LPS treatment resulted in sharing of 72% (61 out of 85 possible) of DEG. A different trend was observed for shared genes responsive to heat treatment with only 32% (7 out of 22 possible). The largest number of shared genes between breeds is for the LPS+heat treatment, 399 DEG (out of a possible 568, corresponding to 70%).

### GO enrichment analysis

**Fig 4:**
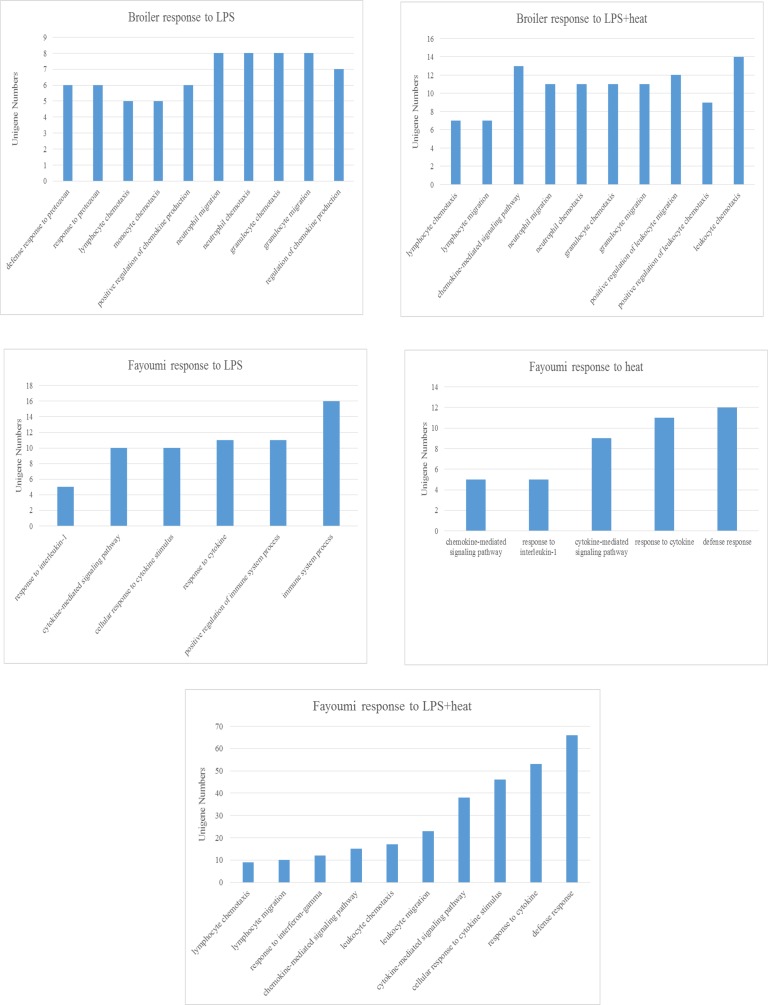
Gene ontology (GO) enrichment analyses showing top significantly associated GO terms based on differentially expressed genes. Fayoumi and broiler chicken breeds at 22 days of age after 7 hours of heat treatment at 35°C, and/or 3.5 hours post lipopolysaccharide (LPS) treatment. All enrichments had a p value ≤ 0.05.

The DEG from each of the contrasts (6 total) were used for GO enrichment analysis (www.geneontology.org) for biological process enrichment and the top ten enrichments are shown in Fig 4.

### Validation of RNA-sequencing data using fluidigm technology

**Fig 5:**
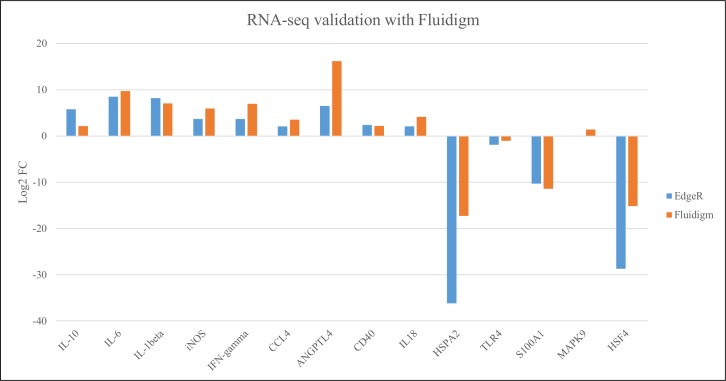
Validation of RNA-sequencing with Fluidigm technology. A total of 32 RNA samples (4 per treatment) were assayed in triplicate on a FlexSix IFC where we tested mRNA expression of 14 (IL-10, IL-6, IL-1beta, iNOS, IFN-gamma, CCL4, ANGPL4, CD40, IL-18, HSPA2, TLR4, S100A1, MAPK9, and HSF4) test genes and 2 housekeeping genes. Log_2_ fold change determined using EdgeR with RNA-sequencing (blue bars) and Fluidigm technology (orange bars). The correlation between the two technologies was (R = 0.86).

Validation of the RNA-sequencing data was completed using Fluidigm gene expression technology. A total of 32 RNA samples (4 per treatment) were assayed in triplicate on a FlexSix IFC where we tested mRNA expression of 14 test genes and 2 housekeeping genes. The comparison of fold changes calculated from Fluidigm and RNA-sequencing are depicted in Fig 5. The correlation between the two technologies was (R^2^ = 0.84).

### Ingenuity pathway analysis and principle component analysis

All DEG from each contrast were used for input into Ingenuity Pathway Analysis (IPA) software. The results of top canonical pathways are shown in Table 2 for each of the contrast groups. The top predicted upstream regulators are shown in Table 2. All genes with FDR ≤ 0.05 were included in the pathway analysis. The resulting number of genes input and annotated within IPA for each treatment group are: F_HS_PBS had 79 annotated genes of 116 total, F_TN_LPS had 66 annotated genes of 85 total, F_HS_LPS 2925 annotated genes of 3707 total, Br_HS_PBS had 18 annotated genes of 25 total, Br_TN_LPS had 264 annotated genes of 346 total, and Br_HS_LPS had 798 annotated genes of 971 total.

**Table 2:** Ingenuity pathway analysis results for differentially expressed genes due to treatment within breed. The numbers included in the table are P values. The top 5 significant (P ≤ 0.05) canonical pathways, upstream regulators, and diseases and disorders are listed for each contrast.

	**Fayoumi response to heat**		**Broiler response to heat**	
Canonical pathways	Acute Phase Response Signaling	2.02E-07	Lipid Antigen Presentation by CD1	2.12E-02
	Extrinsic Prothrombin Activation Pathways	2.60E-05	Remodeling of Epithelial Adherens Junctions	5.46E-02
	Intrinsic Prothrombin Activation Pathway	1.64E-04	Macropinocytosis Signaling	5.46E-02
	Retinoate Biosynthesis I	2.42E-04	Fcγ Receptor-mediated Phagocytosis in Macrophages and Monocytes	7.40E-02
	Coagulation System	2.89E-04	Paxillin Signaling	8.01E-02
Upstream Regulators	TNF	2.81E-13	CYTH2	7.37E-04
	Lipopolysaccharide	2.37E-12	IPCEF1	7.37E-04
	IKBKB	3.09E-12	SUV39H2	1.47E-03
	IL1B	5.63E-12	LMCD1	1.47E-03
	GH1	1.31E-10	CYTH3	1.47E-03
Diseases and Disorders	Cardiovascular Disease	3.68E-03	Connective Tissue Disorders	8.24E-04
	Developmental Disorder	3.68E-03	Inflammatory Response	8.24E-04
	Hematological Disease	3.68E-03	Skeletal and Muscular Disorders	8.24E-04
	Hereditary Disorder	3.68E-03	Infectious Diseases	4.12E-02
	Immunological Disease	3.68E-03	Cancer	1.39E-02
	**Fayoumi response to LPS**		**Broiler response to LPS**	
Canonical Pathways	Atherosclerosis Signaling	2.58E-06	Altered T Cell and B Cell Signaling in Rheumatoid Arthritis	1.30E-08
	LXR/RXR Activation	3.99E-05	Role of Macrophages, Fibroblasts and Endothelial Cells in Rheumatoid Arthritis	3.94E-08
	Role of Macrophages, Fibroblasts and Endothelial Cells in Rheumatoid Arthritis	3.99E-05	IL-10 Signaling	2.08E-06
	Glucocorticoid Receptor Signaling	2.27E-04	Atherosclerosis Signaling	3.68E-06
	Granulocyte Adhesion and Diapedesis	2.39E-04	IL-17A Signaling in Fibroblasts	4.33E-06
Upstream Regulators	TNF	4.39E-22	TNF	3.22E-51
	Lipopolysaccharide	1.48E-15	Lipopolysaccharide	2.60E-45
	TLR3	1.70E-13	IL1B	2.04E-36
	IL1B	1.55E-12	NFkB (complex)	3.68E-31
	Prostaglandin E2	1.56E-12	IFNG	1.25E-24
Diseases and Disorders	Organismal Injury and Abnormalities	3.15E-03	Inflammatory Response	2.88E-06
	Inflammatory Response	3.15E-03	Organismal Injury and Abnormalities	2.74E-06
	Cancer	2.91E-03	Cancer	2.62E-06
	Gastrointestinal Disease	3.15E-03	Inflammatory Disease	2.09E-06
	Metabolic Disease	3.15E-03	Infectious Diseases	1.72E-06
	**Fayoumi response to heat+LPS**		**Broiler response to heat+LPS**	
Canonical Pathways	Hepatic Fibrosis/Hepatic Stellate Cell Activation	2.58E-10	Hepatic Fibrosis/Hepatic Stellate Cell Activation	5.77E-09
	Axonal Guidance Signaling	1.96E-09	Role of Macrophages, Fibroblasts and Endothelial Cells in Rheumatoid Arthritis	2.71E-08
	Aryl Hydrocarbon Receptor Signaling	7.49E-06	Granulocyte Adhesion and Diapedesis	2.29E-07
	Role of Macrophages, Fibroblasts and Endothelial Cells in Rheumatoid Arthritis	8.83E-06	LPS/IL-1 Mediated Inhibition of RXR Function	2.57E-07
	Glutamate Receptor Signaling	9.87E-06	P38 MAPK Signaling	3.78E-07
Upstream Regulators	TNF	2.89E-46	TNF	5.41E-51
	TGFB1	6.78E-41	Lipopolysaccharide	1.97E-42
	TP53	1.71E-40	IL1B	8.35E-35
	Lipopolysaccharide	7.86E-34	TGFB1	2.12E-34
	Vegf	4.07E-28	TP53	1.42E-27
Diseases and Disorders	Cancer	1.38E-09	Inflammatory Response	3.82E-07
	Organismal Injury and Abnormalities	1.38E-09	Cancer	4.76E-07
	Gastrointestinal Disease	1.12E-09	Organismal Injury and Abnormalities	4.76E-07
	Hepatic System Disease	4.20E-12	Tumor Morphology	7.94E-09
	Reproductive System Disease	2.74E-11	Connective Tissue Disorder	4.74E-07

A principle component analysis was completed in JMP (SAS Institute, Cary, NC, USA) using all count data from all treatment groups and samples that were used for RNA-seq (S2 Fig). The principle component 1 explained 16% of the variation and corresponds to LPS stimulation status (15/15 LPS stimulated birds greater than 0). Whereas 2 explained 44% of the variation and largely corresponds to breed (10/16 Fayoumis greater than 0).

## Discussion

For the first time, to our knowledge, we describe the birds’ phenotypic responses and the changes in the spleen transcriptome in response to thermal treatment (heat stress) and immune stimulation (LPS) alone, and in combination (LPS+heat), in unique lines of chickens. We hypothesized that the lines would differ in their response to these treatments, and that the response of the heat-tolerant and disease-resistant Fayoumis would give insights into the genetic mechanisms of favorable response to these stressors. The Fayoumis were imported to the U.S. from Egypt more than 60 years ago due to their disease resistant nature, and since have been highly inbred >99.9% [28]. Because the Fayoumi breed originated in a region that has a high-temperature climate, this breed has undergone natural selection for tolerance to heat. In contrast, the broilers were commercially selected for muscle mass accretion in temperate climates and were not deliberately inbred [27]. Because of the commercial selection for rapid and efficient growth, we hypothesize the broilers will be less tolerant to heat than the Fayoumis. Studies of an advanced intercross between the Fayoumi and broiler lines determined that body temperature, body weight, breast yield, digestibility [29], and blood components [30] measured during heat stress were heritable and QTL were identified, which further illustrates the heat-response divergence of these two lines. In addition to divergence for heat tolerance, these lines have been characterized for differences in response to challenge with *Salmonella Enteritidis*, which show the Fayoumi to be more resistant than the broiler [20, 31]. Therefore, the Fayoumi and broiler serve as a model for heat and disease differences.

### Phenotypic responses

To characterize the phenotypic response to LPS, heat, and LPS+heat, we measured body temperature and 13 different blood chemistry components. Body temperature increased to the same amount with LPS or heat treatment alone in both breeds. When birds were treated with LPS+heat, the body temperature increased synergistically i.e. beyond the individual treatments. The mean normal body temperature of chickens is 41°C and the highest productivity is within the thermoneutral temperature zone, which has been estimated between 20–26°C for adult broilers and layers [32, 33, 34]. The upper critical temperature is defined as the ambient temperature in which the animal continually increases body temperature until mortality results, and this is estimated between 36–37°C for broilers [35]. Our experiment used an acute heat stress of 7 hours at 35°C. LPS is a well characterized inflammatory stimulus but the effect on body temperature varies considerably depending on breed, age, and exposure time [36, 37, 38, 39, 40]. We based the amount of LPS injected (100 ug/kg of body weight) on previous studies conducted in our lab using the broiler and Fayoumi breeds. demonstrated that subcutaneous injection of LPS at a concentration of 100 ug/kg of body weight induced significant differential gene expression of pro-inflammatory and anti-inflammatory cytokines in the spleens of broilers at 3 hours post-injection [41]. Additionally, Casebere (2015) reported differential gene expression of pro-inflammatory cytokines in white blood cells from Fayoumi and broilers after the same amount of LPS injection [42]. Generally, these reports are consistent that a period of hypothermia precedes hyperthermia. Here we report that at 3.5 hours post injection a significant increase in body temperature was observed, with Fayoumis exhibiting a higher body temperature than broilers. Little is known about the effect of heat stress on immune response. In mice, heat treatment alone does not affect serum levels of TNF-α, IL-1, IL-1B, and acute phase proteins, whereas LPS+heat results in higher concentrations of these inflammatory cytokines compared to LPS alone [43]. However, this result does not necessarily support an improved or reduced immune response, only that more inflammatory molecules are present. If inflammation is not strictly controlled, it may have detrimental results on the host. Additionally, because we observed a synergistic increase in body temperature when the double stimulus (LPS+heat) was done, this could be detrimental to the host.

Blood chemistry components were measured that relate to acid-base balance, electrolytes, and glucose. During periods of heat stress, chickens increase the depth and frequency of breaths [44], which results in alkalosis. Indeed, the current study observed changes related to respiratory alkalosis including increased pH, increased pCO_2_, and increases in TCO_2_. When birds were treated with LPS, the changes in blood components appears to be opposite compared to heat treatment. It may be that the chickens do not attempt to decrease body temperature by respiration in response to an inflammatory stimulus, because inflammation and fever in response to an immune challenge is an efficient method to kill the infectious agent. The changes in blood chemistry components related to acid base balance (pH, BE, HCO_3,_ and TCO_2_) were more similar in response to LPS and to LPS+heat, than to heat alone. We hypothesize that, under heat stress, the birds may not be attempting to decrease body temperature when faced with an immune stimulant. This is reflected in the blood chemistry components of the LPS+heat treatment resembling LPS treatment more than heat treatment. The most significant differences in blood components between breeds was due to treatment with LPS+heat. These blood components may be good biomarkers for combined heat tolerance and disease resistance.

### Spleen transcriptomic responses

#### Heat stress

Heat stress resulted in a larger number (N = 107) of DEGs in Fayoumis compared to broilers (N = 22) in the spleen, and most DEGs in each breed were upregulated. We observed DEGs involved in pathways related to intestinal permeability although the spleen was the tissue investigated. We may have observed these changes in the immune organ due to the crucial function of the spleen to sample products within the blood and because changes in intestinal permeability can alter level of exposure of splenocytes to gut-derived bacteria and their products, such as LPS. During periods of heat stress, intestinal permeability increases in chickens. Broilers under heat stress conditions increase intestinal permeability [3, 8, 15], and layers have altered gut morphology of microvilli [11]. The observed transcriptomic changes in the current study are associated with altered epithelial barriers. The data presented here indicates that broilers may be more susceptible to disruptions in tight junctions of the intestinal mucosa (“leaky gut”). In particular, we observe changes in pathways such as “Remodelling of Epithelial Adherens Junctions” and “Paxillin Signalling”. Interestingly, LPS is identified as an upstream regulator due to heat treatment in Fayoumis. This may be due to leaky gut during heat treatment, which in turn activates the proinflammatory immune response. We observed Acute Phase Response (APR) Signalling as the top canonical pathway in Fayoumi. The APR is known as the extreme change in plasma proteins due to systemic inflammation [45], and functions to restore homeostasis by non-specific immune mechanisms [46]. Typically, acute phase proteins are activated during infection, but we observed an increase during heat stress likely because of their pleiotropic effects. In humans, for example, acute phase proteins increase angiogenesis [47], which could help reduce body temperature. The Fayoumis have a response to heat stress that is largely affecting clotting in the blood. Clotting could reduce permeability of the intestine during heat stress and thus minimize the occurrence of leaky gut syndrome. A DEG of interest in the Fayoumi in response to heat stress was SOCS2 (FC = 2), a suppressor of cytokine signalling. The SOCS2 gene was previously reported as a candidate gene for a very large QTL for breast muscle yield during heat stress in an intercross line between Fayoumi and broiler [29]. Thus, this gene may represent a bridge among traits of heat tolerance, growth and immune response.

#### LPS

The broiler responded to LPS treatment with many more (N = 283) DEG than the Fayoumi (N = 85). The unresponsiveness in the spleen to LPS in the Fayoumi may be due to the effects of anti-inflammatory gene expression. An example of this in the current study is the upregulation of IL10R, which in turn promotes IL10 signalling resulting in immunosuppression by inhibition of transcription of proinflammatory cytokines [48]. A pathway that was strongly (P = 2.27E-04) activated in the Fayoumi in response to LPS treatment was “Glucocorticoid Receptor Signalling”. Glucocorticoids are a class of corticosteroids that are highly conserved across all vertebrates [49], and are involved in reducing inflammation [50]. In a previous study on chickens, LPS increased serum levels of corticosterone [51], which is a major glucocorticoid in chickens and the plasma concentration used as a measure of stress [52]. Heat stress is known to increase corticosterone concentrations in broilers during heat stress [53], although we did not observe it as a top canonical pathway in either breed due to heat treatment. The IL-1RA and IL-1R2 were both upregulated in the current study in response to LPS, and both receptors are involved in the anti-inflammatory response [54]. Another top canonical pathway identified in Fayoumis in response to LPS was Granulocyte Adhesion and Diapedesis. During pathogen invasion, immune cells travel to the site of infection by expressing adhesion proteins and moving from circulation to the infected tissues. An important type of granulocyte in chicken are referred to as heterophils. One study demonstrated that chickens with a reduced (3 to 9 fold) heterophil number have reduced ability to control *Salmonella enteritidis* disease pathogenesis [55]. The ability to activate heterophils during inflammation is desirable. The broiler responded strongly to LPS treatment by inflammatory response. A top pathway in both breeds is Role of Macrophages, Fibroblasts and Endothelial Cells in Rheumatoid Arthritis, which is a disease characterized by uncontrolled inflammation, and we observed upregulation of genes such as IL-1beta, IL-6, and IL-22 which are pro-inflammatory cytokines. The IL-17 pathway was activated in response to LPS in the broiler, and this pathway is known to be activated by NFkB which was upregulated in the current study [56]. The broilers may be increasing inflammation during challenge with LPS potentially leading to uncontrolled inflammatory response.

#### LPS+heat

During stimulation with LPS+heat, several shared pathways were identified between broiler and Fayoumi, such as “Hepatic Fibrosis/Hepatic Stellate Cell Activation” and “Role of Macrophages, Fibroblasts and Endothelial Cells in Rheumatoid Arthritis”. The latter pathway was also identified in both breeds with LPS as the only stimulus, illustrating the important role of inflammatory response pathways to LPS under both thermoneutral and hot temperatures. The pathway related to hepatic function was not identified in either single stimulus. During periods of heat stress in chickens, the liver is sensitive to oxidative damage [57], and the liver is also a known site for bacterial pathogenesis [58]. It may be that the double stressor of heat and LPS is causing fibrosis in the spleen which can be detected in transcriptome changes. After LPS+heat in the Fayoumi, the Aryl Hydrocarbon Receptor Signalling pathway was effected, which is important for immunological responses and inhibiting inflammation [59]. This is thought to occur through upregulation of IL-22 [60]. Most of the Fayoumi’s DEG in response to LPS+heat were downregulated. The broiler showed the effected pathway “LPS/IL-1 Mediated Inhibition of RXR Function” which is thought to lead to impaired metabolism [61], potentially a mechanism to reduce body heat generation.

## Conclusions

This is the first report of the spleen transcriptomic response in unique chicken line to heat stress, LPS, and the double stimulation. Heat and LPS treatment increased body temperature to the same amount, and the double stimulation synergistically increased body temperature. However, blood chemistry components revealed distinct physiological responses to heat and LPS. Furthermore, LPS+heat resulted in a similar blood chemistry response to LPS alone. The Fayoumi had more DEGs in response to heat and LPS+heat than the broiler and the broiler responded with more DEG with LPS. For the first time a thorough investigation into the transcriptomic response to heat stress and immune stimulation has been reported in chickens. A strength of the study included the unique genetic lines (Fayoumi and broiler) to compare responses to the dual stressor (heat stress and LPS). The genes and pathways identified in the study may serve as candidate genes for further investigation into the ability to breed simultaneously for both disease and heat resistance in chickens. Of particular interest are the suggested roles of acute phase proteins, granulocytes and macrophages in modulating the response of birds to stressors.

## Supporting information

S1 Table Primers used for validation of RNA-seq results. Forward and reverse primer sequences used with Fluidigm to validate RNA-sequencing results.
(XLSX)

S1 Fig Experimental Design. The experimental timeline, chicken breeds, and experimental groups (N = 4/group) are displayed.
(PDF)

S2 Fig Principal Component Analysis for RNA-seq Data. A PCA plot based on correlation from expression data. Breed (principle component 1) and LPS stimulation (principle component 2 identifiers colored in blue) largely explain the variation in expression data. Data points are presented according to experimental group with the following identifiers: Fayoumi, TN, PBS as closed circles; Fayoumi, HS, PBS as closed squares; Fayoumi, TN, LPS as closed triangles; Fayoumi, HS, LPS as closed diamonds; Broiler, TN, PBS as empty circles; B, HS, PBS as empty squares; Broiler, TN, LPS as empty triangles; Broiler, HS, LPS as empty diamonds.
(PDF)
