# Supplementary material for: Unique genetic responses revealed in RNA-seq of the spleen of chickens stimulated with lipopolysaccharide and short-term heat
Source: PLoS One. 2017 Feb 6;12(2):e0171414. doi: 10.1371/journal.pone.0171414 (PMC5293231; doi:10.1371/journal.pone.0171414)
Supplement: S1 Fig — The experimental timeline, chicken breeds, and experimental groups (N = 4/group) are displayed. (PDF) [file pone.0171414.s002.pdf]

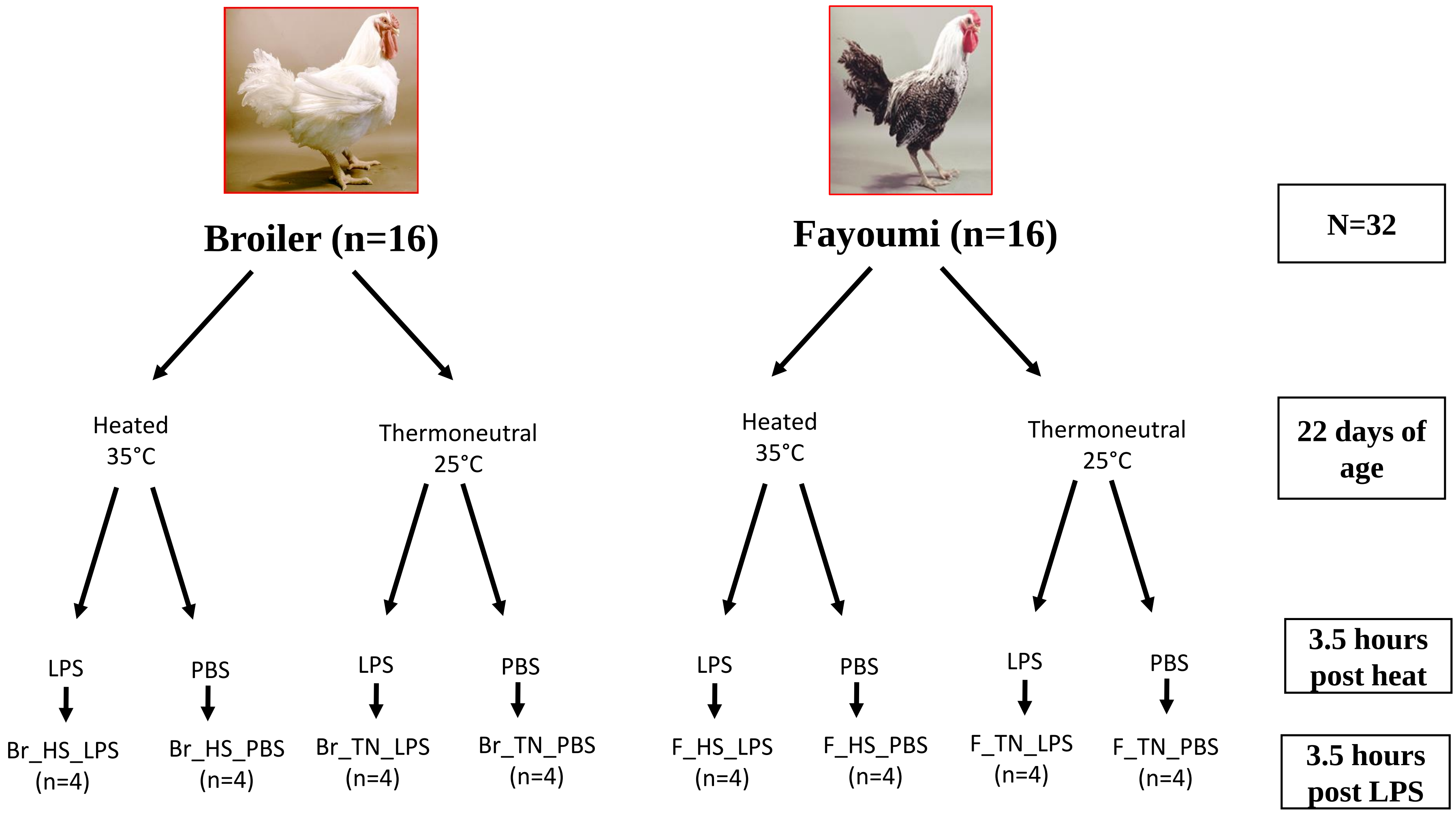

Broiler (n=16)
Fayoumi (n=16)
N=32
Heated
35°C
Thermoneutral
25°C
Heated
35°C
Thermoneutral
25°C
22 days of age
LPS
PBS
LPS
PBS
LPS
PBS
LPS
PBS
3.5 hours post heat
Br_HS_LPS (n=4)
Br_HS_PBS (n=4)
Br_TN_LPS (n=4)
Br_TN_PBS (n=4)
F_HS_LPS (n=4)
F_HS_PBS (n=4)
F_TN_LPS (n=4)
F_TN_PBS (n=4)
3.5 hours post LPS
