# Supplementary figures and images for: Unique genetic responses revealed in RNA-seq of the spleen of chickens stimulated with lipopolysaccharide and short-term heat
Source: PLoS One. 2017 Feb 6;12(2):e0171414. doi: 10.1371/journal.pone.0171414 (PMC5293231; doi:10.1371/journal.pone.0171414)

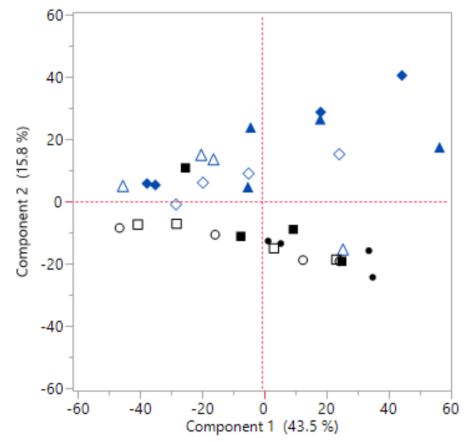

Supplement: S2 Fig — A PCA plot based on correlation from expression data. Breed (principle component 1) and LPS stimulation (principle component 2 identifiers colored in blue) largely explain the variation in expression data. Data points are presented according to experimental group with the following identifiers: Fayoumi, TN, PBS as closed circles; Fayoumi, HS, PBS as closed squares; Fayoumi, TN, LPS as closed triangles; Fayoumi, HS, LPS as closed diamonds; Broiler, TN, PBS as empty circles; B, HS, PBS as empty squares; Broiler, TN, LPS as empty triangles; Broiler, HS, LPS as empty diamonds. (PDF) [file pone.0171414.s003.pdf]
